# Posttraumatic Intradiploic Leptomeningeal Cyst: A Rare Complication of Head Trauma

**DOI:** 10.1155/2015/395380

**Published:** 2015-10-19

**Authors:** Jernailsingh Bava, Ashank Bansal, Santosh Bhaugaunda Patil, Kiran Ashok Kale, Anagha Rajiv Joshi

**Affiliations:** Department of Radiology, Lokmanya Tilak Municipal Medical College and General Hospital, Sion West, Mumbai 400022, India

## Abstract

Posttraumatic intradiploic leptomeningeal cyst is an exceedingly uncommon complication of skull fracture in childhood with only about twenty-one cases described in literature till now. We report 2 such cases of intradiploic leptomeningeal cyst of occipital bone in two 17- and 21-year-old males presenting with headache with history of occipital bone fracture in childhood and briefly discuss its pathogenesis and differential diagnosis.

## 1. Case Report 1

**Figure 1:**
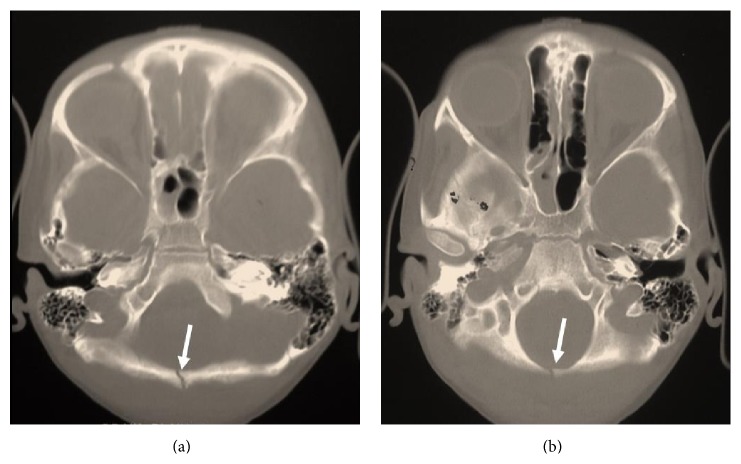
Axial MDCT bone window showing linear undisplaced fracture of occipital bone at the age of 12 years (arrows).

A 17-year-old male presented with history of intermittent headache in the occipital region for 12 months, not relieved by medications. Patient also noticed a swelling in the occipital region which was progressively increasing over a period of 2 years. On examination, an ill-defined smooth hard lump was felt in the occipital region; however the rest of the physical and neurological examinations were normal. Patient gave a past history of head trauma at the age of 12 years due to fall from staircase. A CT scan of head performed at that time revealed linear undisplaced fracture in the midline involving both the inner and outer tables of the occipital bone (Figures 1(a) and 1(b)). The injury was managed conservatively at that time.

**Figure 2:**
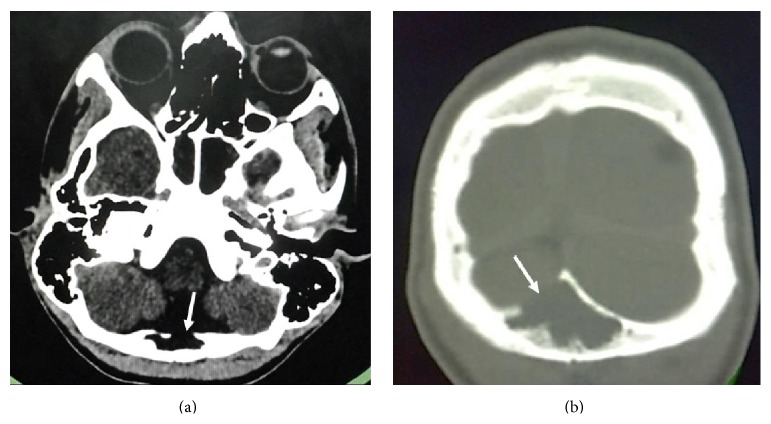
Axial MDCT 5 years later: (a) soft tissue window and (b) bone window showing CSF density lesion in the diploic space of the occipital bone with a break in the continuity of the inner table and expansion of the diploic space (arrows).

**Figure 3:**
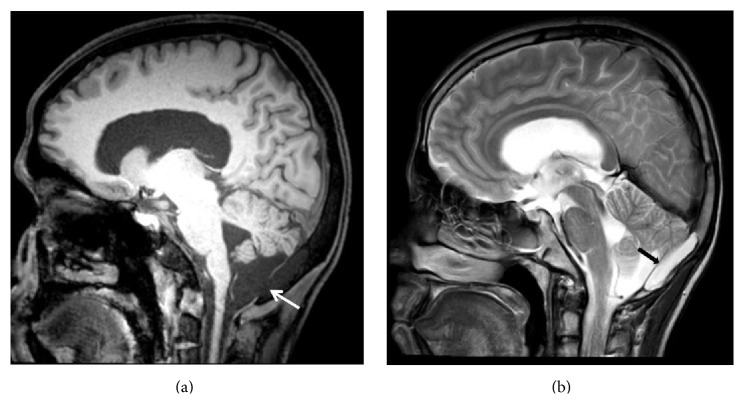
MRI brain (a) T1 sagittal and (b) T2 sagittal images showing intradiploic space expansion with CSF intensity lesion (black arrow) communicating with the cistern magna through a dural tear (white arrow).

Present CT brain revealed a CSF density collection in the diploic space of the occipital bone with a break in the continuity of the inner table and expansion of the diploic space (Figures 2(a) and 2(b)). Magnetic Resonance Imaging (MRI) of brain corroborated the CT findings with MRI revealing intradiploic space expansion with a fluid filled lesion which was following CSF intensity on all sequences (Figures 3(a) and 3(b)). This lesion was seen communicating with the cisterna magna through a dural tear (Figures 3(a) and 3(b)). No restriction of diffusion on DWI sequence was noted. Thus, a diagnosis of posttraumatic intradiploic leptomeningeal cyst was made. Mild communicating hydrocephalus was also noted and patient was referred to the neurosurgery department for surgical repair.

## 2. Case Report 2

**Figure 4:**
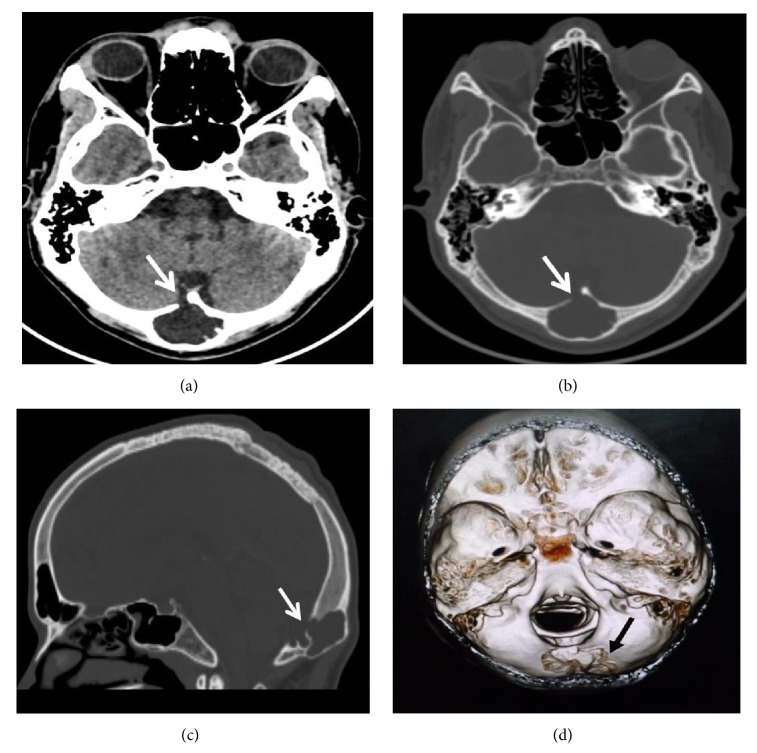
MDCT head (a) axial soft tissue window, (b) axial bone window, (c) sagittal bone window, and (d) VRT images showing CSF density collection in the diploic space of the occipital bone with expansion of the diploic space (arrows).

A 21-year-old male with a past history of trauma 10 years back which was managed conservatively presented in neurology OPD with history of headache for 5 months. No significant finding was noted on clinical examination. As the pain was not getting relieved on standard medications, patient was referred for CT head. CT revealed widening of diploic space of the occipital bone caused by a CSF density lesion which was seen communicating with the cisterna magna through a break in the inner table with scalloping of outer table. No break in outer table was however noted (Figures 4(a)–4(d)).

## 3. Discussion

Posttraumatic intradiploic leptomeningeal cysts are rare variants of growing skull fractures (leptomeningeal cyst). The first case of PTIDLC was reported by Weinand et al. in 1989 [1]. It was also called CSF-diploic fistula [2], intraosseous leptomeningeal cysts [3], posttraumatic growing skull fractures, and posttraumatic arachnoidal cyst. PTIDLCs behave differently from traditional growing fractures and require individualised management decisions.

Posttraumatic intradiploic leptomeningeal cysts are extremely rare complications of calvarial fractures occurring in paediatric patients [4–6]. These cysts are characterized by fracture of inner table and laceration/tear of dura mater with accumulation of CSF in a sac with a covering lined by arachnoid membrane and situated within the diplopic space. Almost all patients give previous history of head trauma with the time interval between trauma and diagnosis of posttraumatic intradiploic leptomeningeal cyst being extremely variable, ranging from 10 months to even 50 years [7–9]. While occipital region is the most common location, PTIDLCs have also been reported in other regions of the skull [7]. Clinical presentation may include headache, ataxia, and occasionally seizures and slow growing swelling.

The most widely agreed-upon hypothesis proposes the herniation of leptomeninges into the intradiploic space through the traumatic rent in the dura mater and inner table. The ball and valve effect due to child's growing brain and the continuous CSF pulsations act as driving expansile forces facilitating the growth of the intradiploic cyst over the years with resultant thinning of the outer table [5, 7, 10]. The thickness of the occipital bone and the thick muscle cover buttressing its outer table explain the predilection of this entity to occur in the occipital region. Other contributory factors in this regard may include the more capacious diploic space as well as cartilaginous origin of occipital bone compared to the membranous origin of the rest of the calvaria [9, 11].

Communicating type of hydrocephalus is often seen to occur along with PTIDLC and may be attributed to the intraventricular haemorrhage at the time of initial trauma [8]. A possibility of subclinical infection leading to hydrocephalus is also to be considered [12].

Common neuroimaging includes a plain radiograph of skull showing eggshell expansion of diploic space with intact outer table. A Computed Tomography (CT) scan of head gives information about the extent of bony defect, intactness of the outer table, and three-dimensional reconstruction aids in surgical planning. Magnetic Resonance Imaging (MRI) of brain is the investigational modality of choice and helps in diagnosis of PTIDLCs by excluding dermoid and epidermoid cyst [8]. In PTIDLCs, signal intensities are similar to CSF on both T1-W image and T2-WI. Associated brain parenchymal pathology, if any, can also be identified.

PTIDLCs need to be differentiated from leptomeningeal cysts (growing skull fractures). The later entity develops as a consequence of diastatic fractures causing laceration of dura mater as well as both the inner and outer tables [10, 13, 14]. These injuries almost always occur in children less than 3 years of age and commonly involve the vertex of the membranous calvaria. Unlike PTIDLCs, porencephalic cysts, cystic encephalomalacia, ipsilateral ventricular dilatation, and seizures are commonly associated with growing skull fractures.

The other differential diagnosis includes intradiploic arachnoid cyst. These are probably congenital in origin and are formed because of obstruction to the flow of CSF from the arachnoid granulations into the venous system. These cysts usually present late in life with local pain, swelling, seizures, or neurological deficit. While radiologically it is difficult to distinguish between the two, a history of trauma and characteristic location in the occipital region favour the diagnosis of PTIDLC.

Surgical management is the mainstay of treatment of PTIDLCs and indications for surgery include large disfiguring swelling and persistent headache [7, 15, 16]. The surgical procedure involves watertight dural closure using duraplasty followed by cranioplasty using autologous split calvarial graft and carries a good prognosis.

## 4. Conclusion

PTIDLC is a rare cause of headache and occurs secondary to calvarial fractures in paediatric age group. Knowledge about the entity is important for the radiologist and clinician as a treatable cause of headache.
